# Theoretical Insight on the Tautomerism and ESIPT Process in Some Hydroxyaryl(hetaryl)idene Azomethine Imines

**DOI:** 10.3390/molecules31020208

**Published:** 2026-01-07

**Authors:** Nikoleta Kircheva, Silvia Angelova, Stefan Dobrev, Liudmil Antonov

**Affiliations:** 1 Institute of Optical Materials and Technologies “Acad. J. Malinowski”, Bulgarian Academy of Sciences, 1113 Sofia, Bulgaria; nkircheva@iomt.bas.bg (N.K.); sea@iomt.bas.bg (S.A.); sdobrev@iomt.bas.bg (S.D.); 2 Institute of Electronics, Bulgarian Academy of Sciences, 1784 Sofia, Bulgaria

**Keywords:** DFT, tautomerism, isomerization, proton transfer

## Abstract

The current study aims to model the potential energy surface (PES) of three much less investigated hydroxyaryl(hetaryl)idene azomethine imine derivatives, possessing the same proton crane unit—namely the azomethine imine moiety—but slightly differing in the structure of the proton transfer platform (stator), by applying the tools of computational chemistry. The obtained calculational results are compared with already reported experimental structural, ^1^H NMR, and UV absorption spectra in an attempt to shed light on the observed data, mainly with regard to the efficiency of the excited state intramolecular proton transfer.

## 1. Introduction

Excited state intramolecular proton transfer (ESIPT), as the abbreviation suggests, is a photochemical process characterized by the translocation of a proton upon photon absorption in the molecule of a compound possessing both proton (hydrogen) donating/accepting groups. Observed and reported for the first time for salicylic acid in 1955 [1], ESIPT has been the main focus of numerous studies aiming to reveal the intimate mechanism of the process [2,3,4,5], as well as to design novel materials with diverse applications as optical sensors [6,7,8], including ESIPT fluorescence-based sensors for cations such as Mg^2+^ [9], Cu^+/2+^ [10], Fe^2+/3+^ [11], anions like pyrophosphate P_2_O_7_^4−^ [12], the difficult-to-detect superoxide O_2_^−^ [13], and small neutral molecules [14,15,16,17,18,19,20,21]. Moreover, by employing the ESIPT process, the activity of different enzymes of great significance for the proper performance of biochemical reactions, e.g., esterases, phosphatases, proteases, and nitroreductases, could be assessed [22,23,24,25,26]. The intriguing field of ultrahigh-density erasable optical memory, attributed to bistable photoswitching of fluorescence emitted by organic molecules, should further be outlined as a plausible application [27,28,29,30,31], along with the area of organic light-emitting diodes (OLEDs) [32,33,34,35].

**Figure 1:**
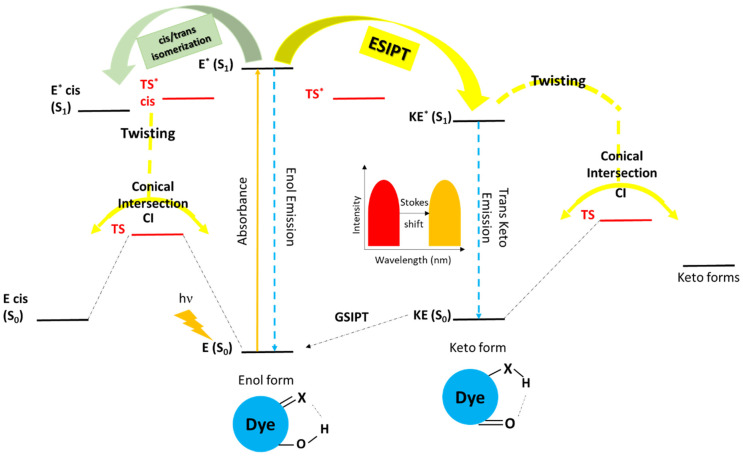
Schematic representation of the ESIPT process in a system with a flexible molecular skeleton.

A typical ESIPT occurs when a chemical compound incorporating in its structure both a hydrogen bond donor, most commonly –OH and in some cases –NH_2_ [36], and an acceptor (usually =N– and C=O) is irradiated with an appropriate wavelength, hence redistributing the electronic charge, which results in increased acidity for the donor and enhanced basicity of the acceptor groups within the initial enol (**E**) form (Figure 1). Note that at the ground state, chromophores typically exist in this particular form, where the distance between the two interacting groups is of immense significance [37,38]. The photoexcitation populates the singlet excited state enol form (denoted as E*). The genuine ESIPT process occurs at this stage, where from the excited state enol form, a proton is transferred to the corresponding excited state keto from (indicated as KE*). This phototautomerization happens on a very short timescale, within femtoseconds (*k*_ESIPT_ > 10^12^ s^−1^) [39]. Afterwards, the KE* undergoes electronic relaxation back to the ground state, being either radiative, characterized by the strongly red-shifted fluorescence designated as the ‘hallmark’ of ESIPT, when the skeleton is rigid as in 10-hydroxybenzo[h]quinoline [40], or non-radiative, most commonly attributed to a trans/cis isomerization reaction (twisting) when the skeleton is flexible [41]. In the former scenario, a full transformation cycle is completed by relocating the proton back to the enol form through the so-called ground state intramolecular proton transfer (GSIPT), whereas the route from the trans-keto form to the cis-keto one is characterized by an energy barrier rendering the process slow. These characteristics allow for experimental measurement of the ESIPT process by fluorescence up-conversion techniques [42,43] or from linewidth information from Shpol’skii matrices [44], as well as the observation of the slower cis/trans isomerization by the nanosecond time-resolved transient difference absorption spectroscopy [45]. It should be noted that, depending on the structure and flexibility of the enol form, trans/cis isomerization is also possible as a process competitive with ESIPT, as observed in some tautomeric azomethine and azo compounds [46,47].

**Figure 2:**
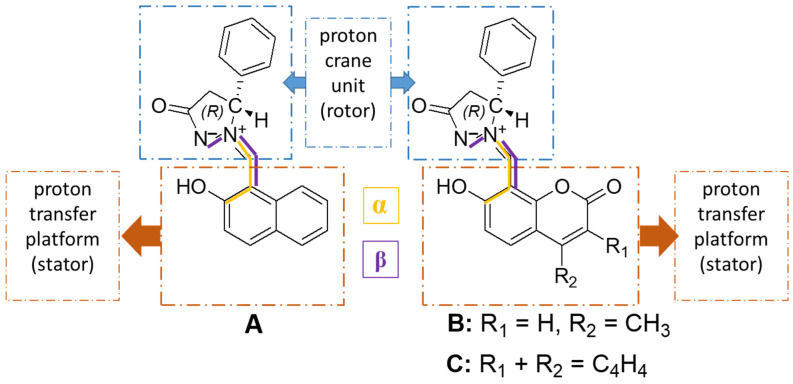
Chemical structures of the compounds under study. Two important dihedral angles α (between atoms C-C-C-N, colored in yellow) and β (between atoms C-C-N-N, colored in purple) are presented. The proton transfer platform (stator), which differs among the compounds, is encircled in brown, whereas the proton crane unit (rotor), which is the same in all cases, is encased in blue.

From a chemical point of view, a great number of compounds fulfill the requirement of possessing a proton donor group in the vicinity of a proton acceptor moiety in combination with a conjugated system, although analogs of 2-(2′-hydroxyphenyl)benzimidazole, 2-(2′-hydroxyphenyl)benzoxazole, and 2-(2′-hydroxy- phenyl)benzothiazole present the most common ESIPT fluorophores [48]. Other ESIPT fluorophores exerting great potential to be noted are those based on quinolone [49], benzophenones [49], flavones [50], anthraquinones [51], and Schiff bases [52,53,54]. The current research focuses on the much less investigated azomethine imine moiety building the structure of three derivatives, namely (**A**) 1-[(2-Hydroxynaphthalen-1-yl)methylidene]-3-oxo-5-phenyl-pyrazolidinium-1,2-ylide; (**B**) (2Z)-2-[(7-Hydroxy-4-methyl-2-oxo-2H-chromen-8-yl)methylidene]-5-oxo-3-phenylpyrazolidin-2-ium-1-ide; and (**C**) (2Z)-2-[(3-Hydroxy-6-oxo-6H-benzo[c]chromen-4-yl)methylidene]-5-oxo-3-phenylpyrazolidin-2-ium-1-ide, presented in Figure 2, and its effect on the tautomerism and the ESIPT process by employing the tools of theoretical chemistry. In these molecules, two fragments can be identified: the proton transfer platform (stator) and the proton crane unit (rotor). The two dihedral angles denoted as α (between the C-C-C-N atoms) and β (between the C-C-N-N atoms), colored in yellow and purple, respectively, in Figure 2, should further be outlined as structural characteristics of great significance for describing the relaxation processes upon irradiation.

The polar 1,3-dipole –N^−^–N^+^=C< fragment in azomethine imines delineates these compounds as indispensable precursors for the preparation of diverse fused heterocycles yielded by cycloaddition reactions [55,56]. On the other hand, they are known for their intriguing spectral properties, like negative photochromism, and their possible application as bistable photochromic compounds [57,58]. Notably, the structures under study have already been synthesized, and their IR, UV absorption, and ^1^H NMR spectra reported [59,60]; hence, the aim of the suggested theoretical approach is to fill in the gaps regarding the mechanism, and the effects of solvent and substituents, which are known to play a crucial role in the excited state intramolecular proton transfer process. Our previous experience with similar ESIPT systems [38,61,62,63] in applying theoretical methods to unveil experimental observables is a solid premise for the reliability of the presented results.

## 2. Results and Discussion

**Figure 3:**
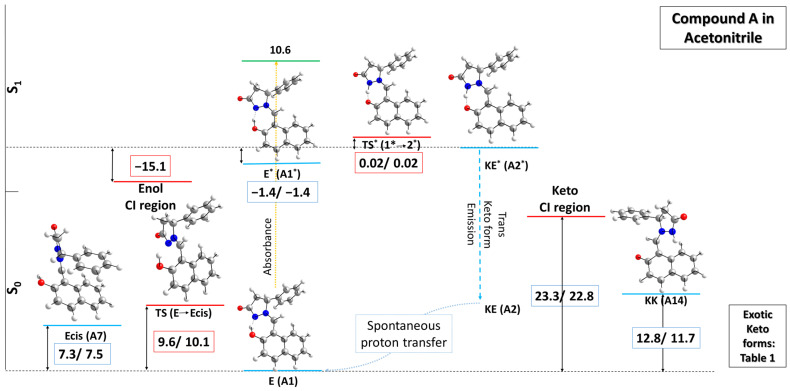
Sketch of the potential energy surface (PES) of compound **A** (presented as R stereoisomer) in acetonitrile in the ground (M062X/TZVP) and first singlet excited (CAM-B3LYP/TZVP) states. The stationary tautomeric forms are indicated in blue, the transition states in red, and the Frank–Condon state in green. The numerical values present relative energy ∆E/relative Gibbs energy ∆G, given in units of kcal mol^−1^. ∆E and ∆G values in the ground and first singlet excited states are calculated with respect to **E** and KE*, respectively. “Enol CI” and “Keto CI” correspond to twisting around the corresponding double bonds (CH=N and C=CH, resp.) in S_1_.

In their possible application as tautomeric molecular switches, the compounds in the current investigation possess the same proton crane unit—namely, the azomethine imine moiety. Their differences, on the other hand, derive from the composition of the proton transfer platform (the conjugated aromatic system containing the OH group): while in compound **A** it is naphthalene, compounds **B** and **C** share the 2-oxo-chromen moiety with diverse substituents at positions 3 and 4. These differences allow not only for distinguishing structural effects on the ESIPT process, but also indicate that the lack of a heteroatom in the second ring leads to a simpler geometry of compound A, rendering proton transfer in this part of the molecule after twisting less probable. All considered initial geometries for structures **A** to **C** are given in Figures S1–S3, respectively. Those found to be the most stable were used to sketch the potential energy surface of the investigated compounds, with the main focus placed on the ESIPT region. First, we report the results obtained for compound **A** in acetonitrile, presented in Figure 3, and in Figure S4 when modeled in toluene as the solvent.

The performed theoretical calculations draw a relatively clear picture of the potential energy surface of compound **A** in the implicit solvent. Not surprisingly, in the ground state it exclusively exists in its enol form, which is found to be the most stable and hence considered a benchmark when comparing all other possible structures in this particular region. Note that two geometries determined by the far asymmetry in the rotor have been taken into account when modeling the enol structure with regard to the dihedral angle α, which differ by about 0.6 kcal mol^−1^ in terms of relative energy when the rotor is positioned either at 32° or −32°, but in both cases secure the necessary proximity of the OH group to the N atom of the pyrazole ring. Upon photoabsorption, the molecule is excited to its first singlet state, given as E*, with a Frank–Condon state substantially high in energy. Interestingly, E* is found to be 1.4 kcal mol^−1^ more stable than the excited keto form (KE*), which, as seen from Figure 3, is taken as the reference structure for the potential energy surface (PES) in the excited state region. This outcome could be attributed to geometrical characteristics such as a shorter O-H bond of 1.04 Å compared to an N-H bond of 1.11 Å, secured by the slightly more planar structure of E* due to a dihedral angle α of −13.3°, as compared to KE* with an α of −16.0°. Additionally, the former is less sterically hindered in the region of the CH axle, where the distance between the two H atoms (one from the axle and the other from the aromatic unit) is 1.95 Å, whereas these atoms are separated by 1.93 Å in the latter. The natural charges in this part of the molecule are affected as well: they change from 0.034 in E* to −0.392 in KE* for the O atom and from −0.205 to 0.170 for the N atom, respectively, due to proton transfer. Moreover, the corresponding transition state from E* to KE* is found to be only 0.02 kcal mol^−1^ higher compared to the excited state keto form. The keto emission to the ground state is expected to directly produce the **E** form, as all performed optimizations of the **KE** form in the ground state lead to spontaneous proton transfer. On the other hand, twisting of the rotor around the CH axle is an alternative path for KE* relaxation, leading to the conical intersection (CI) region. At the corresponding S_0_ transition states, the proton crane unit is positioned perpendicularly to the stator. The energy required for the formation of the two possible transition state (TS) geometries, distinguished by opposing values of α (−91.8° vs. 89.9°), is estimated to be 23.3/22.8 kcal mol^−1^ in terms of relative energy ∆E/relative Gibbs energy ∆G, respectively, for the former structure. These values differ by just about 0.5 kcal mol^−1^ in the latter geometry; therefore, these data are omitted for clarity in Figure 3 and, further, in the other two PES sketches for compounds **B** and **C**. Passing through these transition states yields diverse keto forms, which in the studied compounds can be designated as ‘exotic’ due to their much higher relative energy ∆E/relative Gibbs energy ∆G compared to the most stable E form. The calculated respective ΔE/ΔG values in kcal mol^−1^ of some geometries considered intriguing by the authors are presented in Table 1 for acetonitrile and in Table S1 for toluene.

The calculated ∆E/∆G stand firmly on positive ground, being 18.3/17.6 and 16.5/15.4 kcal mol^−1^ for structures **A3** and **A15**, respectively, implying that the formation of these tautomers is thermodynamically unfavorable. As can be seen from Table 1, where the optimized geometries of these structures are presented, structures **A3** and **A15** correspond to a secondary proton transfer to the carbonyl O atom in the pyrazole ring but twisted around the dihedral α angle at about 190°. Note that the rest of the considered geometries, as shown in Figure S1, could be up to 26 kcal mol^−1^ less stable in terms of ∆G than the enol form (structure **A6**, for example). Therefore, this part of the potential energy surface falls outside the scope of the present research. Since the second ring in compound **A** lacks a heteroatom, no process of subsequent transfer of the proton in this part of the molecule could be observed, unlike in compounds **B** and **C**.

Flexibility, on the other hand, and the existence of a double bond in the connecting moiety between the proton transfer platform and the proton crane unit, on the other hand, give rise to the possibility of an alternative non-radiative pathway attributed to trans/cis isomerization in the modeled enol structures. The corresponding cis stereoisomer of the enol form of compound **A** is found to stand 7.3/7.5 kcal mol^−1^ higher in terms of ∆E/∆G, the route to it defined by two transition states, once again designated to the presence of an asymmetric C atom in the rotor. The performed calculations yielded a transition state from the **E** to the **Ecis** forms that is 9.6/10.1 ∆E/∆G kcal mol^−1^ higher than the most stable structure in the ground state. When excited to S_1_, the Frank–Condon state is found to stand quite high—at 30.2 kcal mol^−1^ in relative energy terms. The relaxed excited singlet state in this part is found to exist in a conical intersection region geometry (twisting around the double C=N bond), almost 15 kcal mol^−1^ lower in energy as compared to the reference KE*. From there on, decay along the side leading to regeneration of the enol form occurs. This non-radiative process competes with ESIPT, decreasing its efficiency [64]. The theoretically observed outcome explains the reported experimental data, as discussed later. It should be noted that an analogous topology of the potential energy surface is drawn for all compounds in the current study due to the retained moieties responsible for the described processes. Therefore, the focus for compounds **B** and **C** falls on the ESIPT region only.

**Figure 4:**
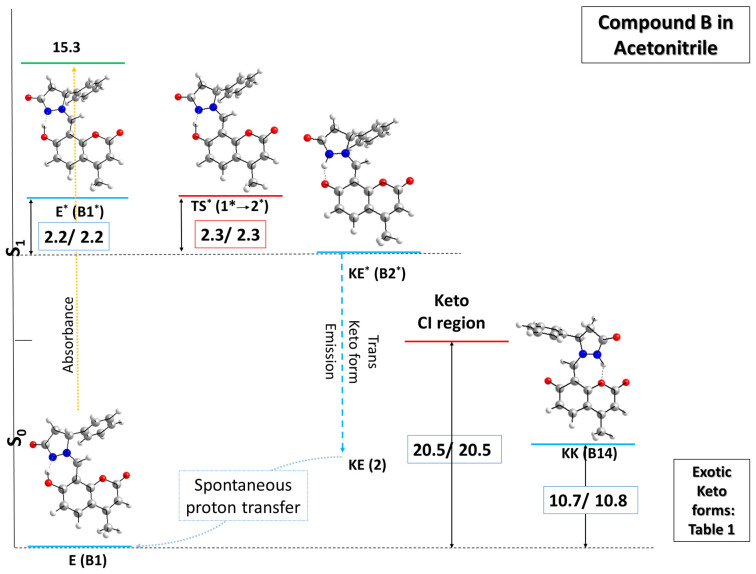
Sketch of the potential energy surface (PES) of model compound **B** (presented as R stereoisomer) in acetonitrile in the ground (M062X/TZVP) and first singlet excited (CAM-B3LYP/TZVP) states. The stationary tautomeric forms are indicated in blue, the transition states in red, and the Frank–Condon state in green. The numerical values present relative energy ∆E/relative Gibbs energy ∆G and are given in units of kcal mol^−1^. ∆E and ∆G values in the ground and first singlet excited states are calculated with respect to **E** and KE*, respectively.

The results obtained for compound B in acetonitrile are further presented in Figure 4, and in Figure S5 for toluene.

The calculations draw a similar sketch of the PES for compound **B** in comparison with compound **A** in the ground state region. The most stable enol form, with an α of −32.6°, an O-H bond of 1.02 Å, and a hydrogen N-H bond distance of 1.56 Å, is again considered as the reference structure in this part of the PES. Its corresponding Frank–Condon and first singlet excited states, however, stand at 15.3 (only ΔE) and 2.2 kcal mol^−1^ higher in terms of both ∆E/∆G, respectively, with respect to the first singlet excited state of the keto form. Additionally, the transition state between the two excited structures falls in the same range of relative energy/relative Gibbs energy, with values of 2.3/2.3 kcal mol^−1^. This difference between compounds **A** and **B,** namely in the ESIPT region, can be assigned to structural characteristics of the E* form of the latter, predisposing the molecule to facilitated proton transfer: the H-atom is bonded to oxygen at 1.06 Å and lies just 1.41 Å away from the nitrogen, with a dihedral angle α of only −0.1°, rendering this structure practically planar and very close to the necessary transition state geometry. Note that the corresponding distances in the E* form of compound **A** are 1.04 and 1.47 Å, with an α of −13.3°. Moreover, the N-H bond in the KE* form of compound **B** is as long as the OH one in the E* form—1.06 Å, whereas the O-H distance now spans 1.52 Å. The dihedral angle almost does not change as well, being calculated to be 1.1°. Bearing in mind that the rotor is the same in both molecules, the observed subtle differences should be ascribed to the proton transfer unit, although no significant change in charge distribution is evidenced by the NBO analysis: shifting from 0.030 in E* to −0.429 in KE* for the O atom and from −0.201 to 0.181 for the N atom, respectively, as a result of proton transfer. Hence, the calculations provide strong evidence for the closeness of E*, KE*, and the transition state connecting them, both in energy and structural characteristics, in the first singlet excited state region, leading to a probable ESIPT process. Again, the ground state keto form of compound **B** is not stable and isomerizes to the enol form. If twisting around the dihedral angle α after relaxation is considered, the corresponding transition state poses quite high relative energy value of 20.5 kcal mol^−1^. The results obtained for subsequent geometries with proton transfer to the carbonyl group in the five-membered ring (structures **B3** and **B15** in Table 1) mirror those calculated for compound **A,** with relative energy ∆E/relative Gibbs energy ∆G values of 17.6/17.6 and 16.6/16.5 kcal mol^−1^, respectively. Moreover, the possibility of transferring the proton to the carbonyl group in the pyran site of the molecule yields a structure denoted as **B19** with 27.4/26.7 kcal mol^−1^ higher in ∆E/∆G.

**Figure 5:**
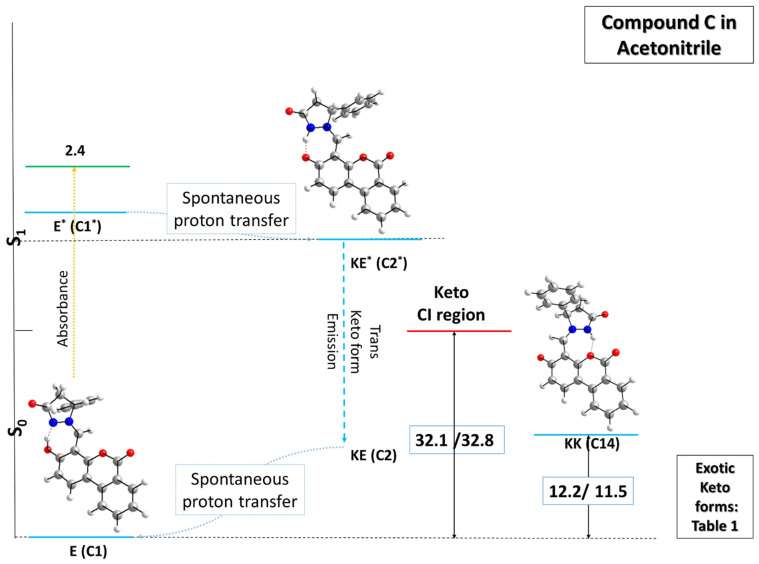
Sketch of the potential energy surface (PES) of model compound **C** (presented as R stereoisomer) in acetonitrile in the ground (M062X/TZVP) and first singlet excited (CAM-B3LYP/TZVP) states. The stationary tautomeric forms are indicated in blue, the transition states in red, and the Frank–Condon state in green. The numerical values present relative energy ∆E/relative Gibbs energy ∆G given in units of kcal mol^−1^. ∆E and ∆G values in the ground and first singlet excited states are calculated with respect to **E** and KE*, respectively.

Finally, the results obtained for sketching the PES of the third modeled compound **C** are depicted in Figure 5 for acetonitrile and in Figure S6 for toluene.

The most stable **E** form of compound **C** resembles to a great extent the previously described compound **B** in its structural characteristics, with an α of 33.5°, an O-H bond length of 1.02 Å, and a hydrogen N-H bond distance of 1.57 Å. Upon photoabsorption, however, the first singlet excited state E* easily transfers the mobile proton from the OH group to the N atom, yielding the KE* form, in which the NH bond is again 1.06 Å, the O-H distance increases to 1.51 Å, and the dihedral angle α is found to be −1.3°. The keto form in the ground state spontaneously transfers the proton back to the most stable enol form. If twisting around the CH axle is considered, the transition state stands 32.1/32.8 kcal mol^−1^ higher in terms of ∆E/∆G than the reference point in the ground state, yielding twisted keto forms similar to those in Table 1 when comparing the compounds in terms of dihedral angle values and relative changes in the calculated relative energy ∆E/relative Gibbs energy ∆G. A slight difference could be found when examining structures 19 for the two compounds. It can be seen that this geometry is about 8 kcal mol^−1^ less stable for compound **C** as compared with compound **B**. This outcome can be attributed to the slightly elongated H-O distance in the former, resulting from the presence of a third aromatic ring engaging the oxygen atom in additional interactions in this part of the molecule. Nevertheless, the structural differences in the stator in compounds **B** and **C** do not affect the overall topology of the potential energy surface of these molecules, as implied by the obtained calculations.

Comparing the results obtained in the two modeled solvents, the stabilizing effect of polar acetonitrile should be outlined. The calculated structures stand closer to the reference enol forms in the ground state, and the energy required to reach the desired transition states is about 2 to 3 kcal mol^−1^ lower. Moreover, in the first singlet excited state, most of the desired structures required for drawing the potential energy surface in the ESIPT region have been successfully optimized, whereas in toluene the mobile proton is much more readily transferred between the forms. This outcome supports the experimental observations, where the most exploited solvent is acetonitrile [59,60].

A final step in the current study involves comparing the results obtained through the applied theoretical approach with the reported experimental data in [57,59,60]. Table 2 summarizes the simulated NMR spectra at two different levels of theory—M062X and B3LYP—in conjunction with the TZVP basis set in both cases.

The experimental spectra of compound **A1** in CDCl_3_ show a methine proton signal in the range of 7.1–7.8 ppm, while the hydroxyl proton resonates at 12.8 ppm. The chemical shifts calculated for the methine proton of **A1** (8.0 ppm at M062X/TZVP and 7.6 ppm at B3LYP/TZVP) and for the hydroxyl proton of the stator unit (12.4 ppm at M062X/TZVP and 11.8 ppm at B3LYP/TZVP) show very good agreement with the experimentally measured values for **A1** in chloroform. This correspondence highlights the reliability of the applied theoretical approach and confirms the existence of the enol form in solution. The keto tautomer, if exists, should be deshielded [46].

Experimental data for **A1** are also available in DMSO-d_6_. In this solvent, the calculations predict either no downfield shift or only a very slight one for the **A1** signals relative to those in CHCl_3_.

For the preferred enol form of compound **B** (**B1**), for which experimental data are available only in DMSO-d_6_, the methine proton is calculated to resonate at 8.0 ppm (M062X/TZVP) and 7.6 ppm (B3LYP/TZVP). The hydroxyl proton is predicted to be shifted downfield relative to **A1**, appearing at 13.6 ppm and 12.9 ppm at the M062X/TZVP and B3LYP/TZVP levels of theory, respectively (Table 2). For C1, again experimentally characterized only in DMSO-d_6_, the methine proton is calculated to resonate at 8.2 ppm (M062X/TZVP) and 7.8 ppm (B3LYP/TZVP), while the hydroxyl proton is predicted to lie at 13.2 ppm and 12.5 ppm at the M062X/TZVP and B3LYP/TZVP levels of theory, respectively (Table 2).

Overall, the chemical shifts calculated for the methine and hydroxyl protons of **B1** and **C1** fall within the same ranges as those of **A1**, supporting the consistency of the computational results and the reliability of the prediction for exclusive presence of the enol tautomer across the series.

**Figure 6:**
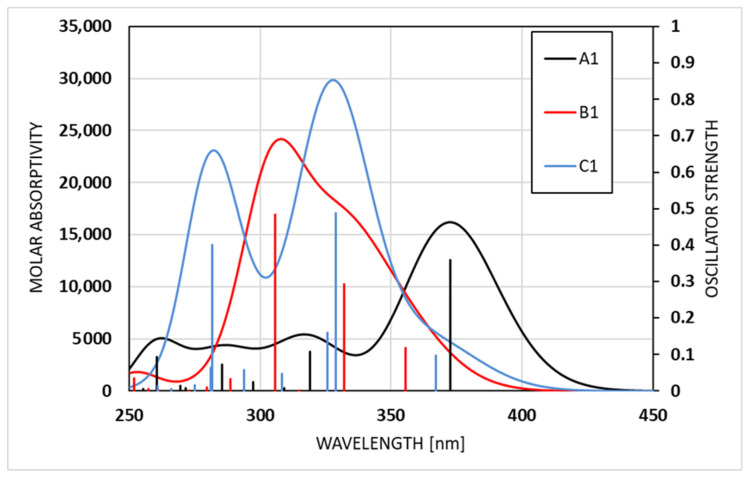
TDDFT-simulated absorption spectra of the compounds under study: enol structures **A1** (black), **B1** (red), and **C1** (blue) in acetonitrile. The vertical lines indicate the oscillator strengths of the corresponding transitions.

The absorption spectra of the preferred enol form of compounds **A**–**C** are shown in Figure 6 and Figure S7 for acetonitrile and toluene, respectively. The overall spectral profiles reflect the Gaussian broadening applied to the discrete calculated transitions, with peak intensities scaled according to the corresponding oscillator strengths. The simulated absorption spectra of **A1**, **B1**, and **C1** on acetonitrile reveal clear differences arising from variations in their electronic structures (Figure 6 and Table 3). A1 shows the most bathochromically shifted band, with a broad maximum at 372 nm, consistent with lower calculated excitation energies and moderate oscillator strengths. Notably, the experimentally observed absorption spectrum of compound **A** exhibits the strongest band at 377 nm [57]. **B1** exhibits two pronounced maxima in the range of 300–350 nm, both associated with higher oscillator strengths (Table 3). The authors of [60] report similar results, with the most pronounced λ_max_ at 324 nm and a shoulder at 389 nm (ε = 24,000 and 5600 L/(cm.M), respectively), which agrees in both shape and intensity with Figure 6. In contrast, as seen from Table 3, **C1** displays the most intense and most hypsochromically shifted simulated absorption, dominated by strong transitions in the 290–310 nm region (experimental λ_max_ at 289 and 336 nm (with a shoulder at 400 nm) [60]), indicative of highly allowed excitations and enhanced electronic delocalization. Overall, as seen from Table 3 and Figure 6, the simulated absorption spectra of the enol forms reproduce the experimental spectra of the compounds under study in very good agreement [59,60], capturing both the overall band shapes and the relative positions and intensities of the main transitions. According to general knowledge of similar azomethine systems, the existence of the keto tautomer should lead to substantially red-shifted absorption maxima [46].

**Figure 7:**
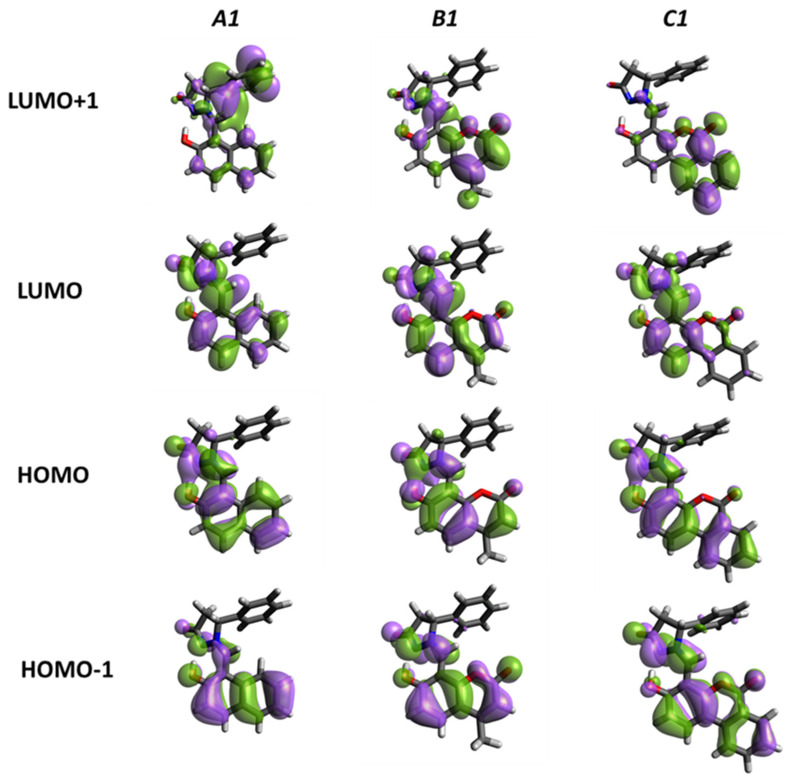
Illustration of frontier molecular orbitals of the preferred enol forms of compounds **A**–**C**. The orbitals are visualized with the Avogadro molecule editor and visualizer. The contour threshold of 0.02 a.u. has been applied.

The TDDFT results (Table 3 in acetonitrile and Table S3 in toluene) show that the low-lying excited states of compounds **A**–**C** in both acetonitrile and toluene are characterized predominantly by π–π* electron transitions involving mainly the frontier molecular orbitals (HOMO, HOMO−1, LUMO, and LUMO+1). The frontier molecular orbitals are mainly distributed over the conjugated π-system of the molecule, with negligible contribution from the substituent groups, as further seen in Figure 7.

For compound **A**, the first excited state is characterized by a strong HOMO→LUMO transition with a relatively high oscillation strength (f = 0.3599) and an excitation energy of 3.33 eV (372 nm). This transition is optically allowed and involves mainly single-orbital excitation. The second excited state involves mixed contributions, mainly from HOMO−1→LUMO, and exhibits a lower but still noticeable oscillatory strength (f = 0.1078). In compound **B**, there are several optically active transitions. The lowest-energy excited state consists of multiple configurations, including HOMO→LUMO and adjacent orbital transitions, resulting in a moderate oscillator strength (f = 0.1178). The second excited state, dominated by HOMO−1→LUMO, shows an increased oscillator strength (f = 0.2923). A higher excited state (excited state 4) shows the highest oscillator strength (f = 0.4829), mainly associated with the HOMO→LUMO+1 transition, suggesting an increased dipole moment of the transition due to increased orbital overlap. For compound **C**, the first excited state is predominantly HOMO→LUMO in nature but has a relatively small oscillator strength (f = 0.0979). In contrast, the second excited state, dominated by HOMO−1→LUMO, shows a pronounced oscillatory strength (f = 0.4883), representing the most intense transition in the low-energy region. Higher excited states include transitions to LUMO+1 and LUMO+2, maintain significant oscillator forces, and show extended π-conjugation.

Similar qualitative trends are observed in toluene, albeit with slight changes in excitation energies and oscillator strengths (see Table S3). For compound **A**, the first excited state remains a strong HOMO→LUMO transition, accompanied by a slight bathochromic shift and an increased oscillator strength (f = 0.3914) compared to acetonitrile. For compound **B**, multiple excited states contribute to the absorption profile. The first and second excited states arise from mixed HOMO/HOMO−1→LUMO transitions with moderate oscillator strengths, while excited state 4 shows the highest intensity (f = 0.5709), mainly due to HOMO→LUMO+1 excitation. This suggests that higher-energy virtual orbitals play an important role in determining the optical response in a less polar solvent. In compound **C**, the first excited state is again predominantly HOMO→LUMO in nature, with moderate oscillator strength. The second and third excited states show mixed orbital contributions and comparable oscillatory strengths, while excited state 6, dominated by HOMO→LUMO+1, shows the highest intensity (f = 0.5040), corresponding to a higher-energy absorption band.

Comparison between acetonitrile and toluene shows that the solvent polarity slightly affects the excitation energies and intensities but does not significantly change the nature of the electronic transitions. The dominant transitions retain their π–π* character in both solvents, while variations in oscillator strengths suggest changes in orbital mixing and dipole moments of the transition caused by the solvent environment.

The predicted excited state PESs shown in Figure 3, Figure 4 and Figure 5 indicate a substantial difference between **A**, on the one hand, and **B** and **C**, on the other hand. In A, the ESIPT process seems to be prohibited, as KE* is less stable compared to E*. This means that the relaxation through the **E** to **Ecis** conical intersection remains the dominant channel. In practical means, this suggests that no ESIPT emission could be detected. According to [55], no emission has been detected for this compound, which nicely confirms our theoretical hypothesis. In the case of **B** and **C**, weak emission has been measured [58] at approximately 540 and 538 nm in acetonitrile. The corresponding Stokes shifts of 7200 and 6400 cm^−1^, respectively, clearly indicate an ESIPT process, as suggested in Figure 4 and Figure 5. Nevertheless, emissionless channels, either through trans-to-cis enol or keto forms, remain the major pathways for relaxation to the ground state in these two compounds.

## 3. Theoretical Methodology

Using the Gaussian 16 suite of programs [65], all necessary calculations were performed without restrictions in the modeled surrounding media, employing tight optimization criteria and an ultrafine grid in the computation of two-electron integrals and their derivatives. The structures under study have been subjected to full geometry optimization at the M062X [66,67] theoretical level in the ground state and at the CAM-B3LYP [68] level for the singlet excited states using the time-dependent DFT procedure [69], both combined with the TZVP basis set [70]. This was followed by vibrational frequency analysis in all cases. The latter indicated local minima on the potential energy surface, as no imaginary frequencies were observed for the obtained optimized geometries. The Polarizable Continuum Model [71], as implemented in Gaussian 16 in its integral equation formalism variant, IEFPCM, was used to implicitly simulate the two solvents, toluene and acetonitrile. Gibbs energy calculations were performed at 298 K and 1 atm using the rigid-rotor harmonic oscillator approximation. We note that this approach may introduce uncertainties for flexible systems with low-frequency vibrational modes; however, relative free energy trends and rotational barriers are expected to be less affected due to error cancelation [72,73]. The version of the NBO analysis integrated into Gaussian 16: NBO 7.0, was implemented to prepare the results discussed in terms of natural bond orbitals. Furthermore, transition states were estimated at the CAM-B3LYP theoretical level in conjunction with the TZVP basis set and verified once more by performing frequency calculations in the modeled medium. Notably, the presence of only one imaginary frequency with a sufficiently high value corresponding to the sought bond-breaking/forming indicated the particular transition state.

The utilized theoretical methodology has been widely verified and applied in previous studies [63,74,75]. First, exploration of the ground state potential energy surface by optimizing tautomeric structures at the M062X theoretical level provides very good predictability in solution. In the case of 8- (benzo[d]thiazol-2-yl)quinolin-7-ol (HQBT), a compound never synthesized or studied before but previously suggested by us [63], it was demonstrated that the results for the excited state PESs obtained by either M062X or CAM-B3LYP are in essence the same. Nevertheless, the latter method was applied in the current study for exploring excited states based on its reported better performance (in comparison with a variety of density functionals, including M062X) in describing valuable characteristics such as electronic excitation energies, excited state geometries, dipole moments, and oscillator strengths across a wide range of systems, including the ESIPT process. Moreover, by comparing DFT results for both ground and excited states in HQBT [68] at the chosen theoretical level with the ‘gold standard’ methods—chemical accuracy-domain-based local pair natural orbital coupled-cluster singles and doubles with perturbative triple excitations (DLPNO-CCSD(T)) and domain-based local pair natural orbital similarity-transformed equation-of-motion coupled-cluster singles and doubles (DLPNO-STEOM-CCSD), respectively—the applied theoretical methodology has been validated and proven accurate. In regard to the respective compounds in the current study, solid evidence for the reliability of the implemented methodology is provided in Table S2, where calculated and experimental (X-ray) parameters for the preferred enol tautomers of compounds **A**–**C** (gas-phase optimized) are compared. The three examined structures show a high degree of geometric consistency, with most C–N, N–N, and C–C bond lengths differing by only a few hundredths of an ångström. A meaningful variation appears in the C7–C8–O2 region, where **A1** exhibits the shortest C7–C8 bond, whereas **B1** and **C1** display slightly shorter C8–O2 distances, indicating a small redistribution of π-electron density. The C(3)-O(1) bond is shorter in all three structures compared with the experimental **A1** value. The intramolecular hydrogen bond H(2)…N(2) is strongest in **B1**, as reflected by the shortest H…N distance. Conformationally, **B1** and **C1** show somewhat smaller dihedral angles in Fragment I, pointing to a more planar arrangement and slightly enhanced conjugation. Overall, the structural differences are subtle but consistent with small variations in electronic delocalization and hydrogen bond strength.

Using the M062X optimized ground state geometries, the UV-Vis spectra were predicted by the B3LYP functional [76] (again in combination with the TZVP basis set). For each structure under study for which a UV spectrum was simulated in the corresponding solvent, six excited states were considered during the calculation. The methodology previously described by us [77] was implemented to simulate the spectra according to a single Gaussian band shape with a half-bandwidth (∆ν_1/2_) of 3000 cm^−1^.

The ^13^C and ^1^H NMR chemical shieldings were computed using the Gauge-Including Atomic Orbitals (GIAOs) method as implemented in Gaussian 16. All calculations were carried out at the density functional theory (DFT) level employing two exchange–correlation functionals, M062X and B3LYP, in combination with the TZVP basis set. To enable direct comparison with the experimental spectra, the calculated absolute shieldings were converted to chemical shifts relative to tetramethylsilane (TMS), according to the following equation:

δ = δ_calc_ (TMS) − δ_calc_

Both δ_calc_ (TMS) and δ_calc_ were obtained using the same functional and basis set to ensure internal consistency. Solvent effects were incorporated by employing the PCM implicit solvation model with acetonitrile as the solvent during geometry optimization and subsequent NMR shielding calculations.

Figure 7 was prepared through the implementation of the Avogadro open-source molecular builder and visualization tool [78,79].

## 4. Conclusions

Excited state intramolecular proton transfer (ESIPT) is an intriguing process with diverse applications in many scientific fields, such as optics. The continuous search for appropriate molecules exhibiting the desired properties has led us to the investigation of hydroxyaryl(hetaryl)idene azomethine imines by utilizing the tools of theoretical chemistry to observe processes at the molecular level. The obtained results indicate a more probable ESIPT on the potential energy surface of compounds **B** and **C** as compared to compound **A**. It was found, however, that the non-radiative trans–cis isomerization provides an alternative relaxation pathway due to the specific structural composition of the stator-binding and rotor moieties retained in all cases. The conclusions drawn regarding, for example, the effect of chemical composition and the nature of the solvent are in good agreement with previously reported data. Simulated ^1^H NMR and UV absorption spectra, in line with experimental ones, further prove the reliability of the results presented in the current research.

## Figures and Tables

**Table 1:** Calculated ∆E/∆G values in kcal mol^−1^ units at the M062X/TZVP theoretical level for obtaining some intriguing/exotic keto forms in acetonitrile.

Forms (See Figures S1–S3)		Optimized Geometry	α/β [°]	∆E	∆G
Compound **A**	**A3**	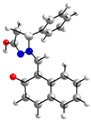	−36.3/ −18.8	18.3	17.6
	**A15**	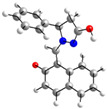	157.6/ −13.1	16.5	15.4
	**A19**	-	-	-	-
Compound **B**	**B3**	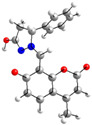	−37.2/ −13.2	17.6	17.6
	**B15**	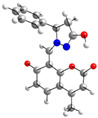	151.4/ −10.4	16.6	16.5
	**B19**	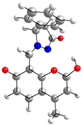	−144.0/1.7	27.4	26.7
Compound **C**	**C3**	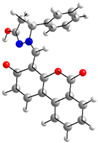	−35.7/ −14.0	18.2	17.9
	**C15**	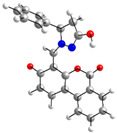	151.5/ −10.6	17.4	16.9
	**C19**	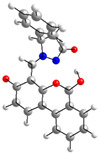	−152.8/2.1	35.7	35.5

**Table 2:** Comparison between experimentally reported ^1^H NMR chemical shifts (in ppm) of selected atoms in compounds **A1** ([57], superscript 1), **B1**, and **C1** ([60], superscript 2), and those calculated at two theoretical levels (geometry of the preferred enol tautomer) in chloroform and DMSO. Underlined atoms indicate the nuclei for which NMR signals are reported.

	Experiment			M062X/TZVP			B3LYP/TZVP		
	A1	B1	C1	A1	B1	C1	A1	B1	C1
Atom	Chloroform								
>CH–	7.1–7.8 ^1^	-	-	8.0	8.0	8.1	7.6	7.6	7.7
OH	12.8 ^1^	-	-	12.4	13.7	13.4	11.8	13.0	12.6
	Dimethyl sulfoxide								
>CH–	7.7–8.4 ^1^	7.8 ^2^	7.9 ^2^	8.1	8.0	8.2	7.7	7.7	7.8
OH	13.1 ^1^	13.7 ^2^	13.7 ^2^	12.4	13.6	13.2	11.7	12.9	12.5

**Table 3:** Calculated excitation energies (E), wavelengths (λ), oscillator strengths (f), and dominant orbital contributions for the low-lying excited states of the preferred enol forms of compounds **A**–**C** in acetonitrile, obtained using TDDFT at the B3LYP/TZVP level of theory. Only excited states with oscillator strengths ≥ 0.1 are reported.

Structure		Transition	Eigenvectors	E, eV	λ, nm	f
**A1**	Excited state 1	83 -> 84 (HOMO -> LUMO)	0.69891	3.3286	372	0.3599
	Excited state 2	80 -> 84	0.13694	3.8878	319	0.1078
		82 -> 84 (HOMO-1 -> LUMO)	0.66338			
		83 -> 88	−0.15625			
**B1**	Excited state 1	90 -> 92	0.23885	3.4878	355	0.1178
		91 -> 92 (HOMO -> LUMO)	0.62379			
		91 -> 93	0.21217			
	Excited state 2	90 -> 92 (HOMO-1 -> LUMO)	0.60418	3.7321	332	0.2923
		91 -> 92	−0.29510			
		91 -> 93	0.18044			
	Excited state 4	90 -> 92	−0.24464	4.0549	306	0.4829
		91 -> 92	−0.11647			
		91 -> 93 (HOMO -> LUMO+1)	0.63385			
**C1**	Excited state 1	99 -> 101	−0.14335	3.3783	367	0.0979
		100 -> 101 (HOMO -> LUMO)	0.67067			
		100 -> 102	0.12421			
	Excited state 2	99 -> 101 (HOMO-1 -> LUMO)	0.66847	3.7694	329	0.4883
		100 -> 101	0.15006			
	Excited state 3	100 -> 101	−0.11603	3.8056	326	0.1586
		100 -> 102 (HOMO -> LUMO+1)	0.68358			
	Excited state 6	97 -> 101	−0.20473	4.4026	282	0.3999
		99 -> 102	−0.15033			
		100 -> 103 (HOMO -> LUMO+2)	0.62810			

## Data Availability

The original contributions presented in this study are included in the article/Supplementary Materials. Further inquiries can be directed to the corresponding author.

## Supplementary Materials

The following supporting information can be downloaded at: https://www.mdpi.com/article/10.3390/molecules31020208/s1, **Figure S1**: Chemical structures considered as initial geometries for the studied compound **A**. Those used for sketching the PES are circled in blue. Their relation to the PES is denoted with the widely accepted designation **E**, **KE**, **KK**, and **K**; Figure S2: Chemical structures considered as initial geometries for the studied compound **B**. Those used for sketching the PES are circled in blue. Their relation to the PES is denoted with the widely accepted designation **E**, **KE**, **KK**, and **K**; Figure S3: Chemical structures considered as initial geometries for the studied compound **C**. Those used for sketching the PES are circled in blue. Their relation to the PES is denoted with the widely accepted designation **E**, **KE**, **KK**, and **K**; Figure S4: Sketch of the potential energy surface (PES) of model compound **A** (presented as R stereoisomer) in toluene in the ground (M062X/TZVP) and first singlet excited (CAM-B3LYP/TZVP) states. The stationary tautomeric forms are indicated in blue, the transition states in red, and the Frank–Condon state in green. The numerical values present relative energy ∆E/relative Gibbs energy ∆G given in units of kcal mol^−1^. ∆E and ∆G values in the ground and first singlet excited states are calculated with respect to **E** and **KE***, respectively; Figure S5: Sketch of the potential energy surface (PES) of model compound **B** (presented as R stereoisomer) in toluene in the ground (M062X/TZVP) and first singlet excited (CAM-B3LYP/TZVP) states. The stationary tautomeric forms are indicated in blue, the transition states in red, and the Frank–Condon state in green. The numerical values present relative energy ∆E/relative Gibbs energy ∆G given in units of kcal mol^−1^. ∆E and ∆G values in the ground and first singlet excited states are calculated with respect to **E** and **KE***, respectively; Figure S6: Sketch of the potential energy surface (PES) of model compound **C** (presented as R stereoisomer) in toluene in the ground (M062X/TZVP) and first singlet excited (CAM-B3LYP/TZVP) states. The stationary tautomeric forms are indicated in blue, the transition states in red, and the Frank–Condon state in green. The numerical values present relative energy ∆E/relative Gibbs energy ∆G given in units of kcal mol^−1^. ∆E and ∆G values in the ground and first singlet excited states are calculated with respect to **E** and **KE***, respectively; Figure S7: TDDFT-simulated absorption spectra of the compounds under study, structures **A1** (black), **B1** (red), and **C1** (blue) in toluene. The vertical lines indicate the oscillator strengths of the corresponding transitions; Table S1: Calculated ∆E/∆G values in units of kcal mol^−1^ at the M062X/TZVP theoretical level for obtaining some intriguing/ exotic keto forms in toluene; Table S2: Calculated and experimental (X-ray) parameters for the preferred enol tautomers of compounds **A**–**C** (gas-phase optimized); Table S3: Calculated excitation energies (E), wavelengths (λ), oscillator strengths (f), and dominant orbital contributions for the low-lying excited states of the preferred enol forms of compounds **A**–**C** in toluene, obtained using TDDFT at the B3LYP/TZVP level of theory. Only excited states with oscillator strengths ≥ 0.1 are reported.
