# Supplementary material for: Theoretical Insight on the Tautomerism and ESIPT Process in Some Hydroxyaryl(hetaryl)idene Azomethine Imines
Source: Molecules. 2026 Jan 7;31(2):208. doi: 10.3390/molecules31020208 (PMC12844085; doi:10.3390/molecules31020208)
Supplement: Supplementary file 1 [file molecules-31-00208-s001.zip › molecules-4045134-supplementary.pdf]

Theoretical Insight on the Tautomerism and ESIPT Process in Some Hydroxyaryl(hetaryl)idene Azomethine Imines

Nikoleta Kircheva [1,2], Silvia Angelova [1], Stefan Dobrev [1] and Liudmil Antonov [2,*]

1 Institute of Optical Materials and Technologies "Acad. J. Malinowski", Bulgarian Academy of Sciences, 1113 Sofia, Bulgaria; nkircheva@iomt.bas.bg (N.K.); sea@iomt.bas.bg (S.A.); sdobrev@iomt.bas.bg (S.D.)

2 Institute of Electronics, Bulgarian Academy of Sciences, 1784 Sofia, Bulgaria

* Correspondence: liudmil.antonov@gmail.com

Supplementary Materials

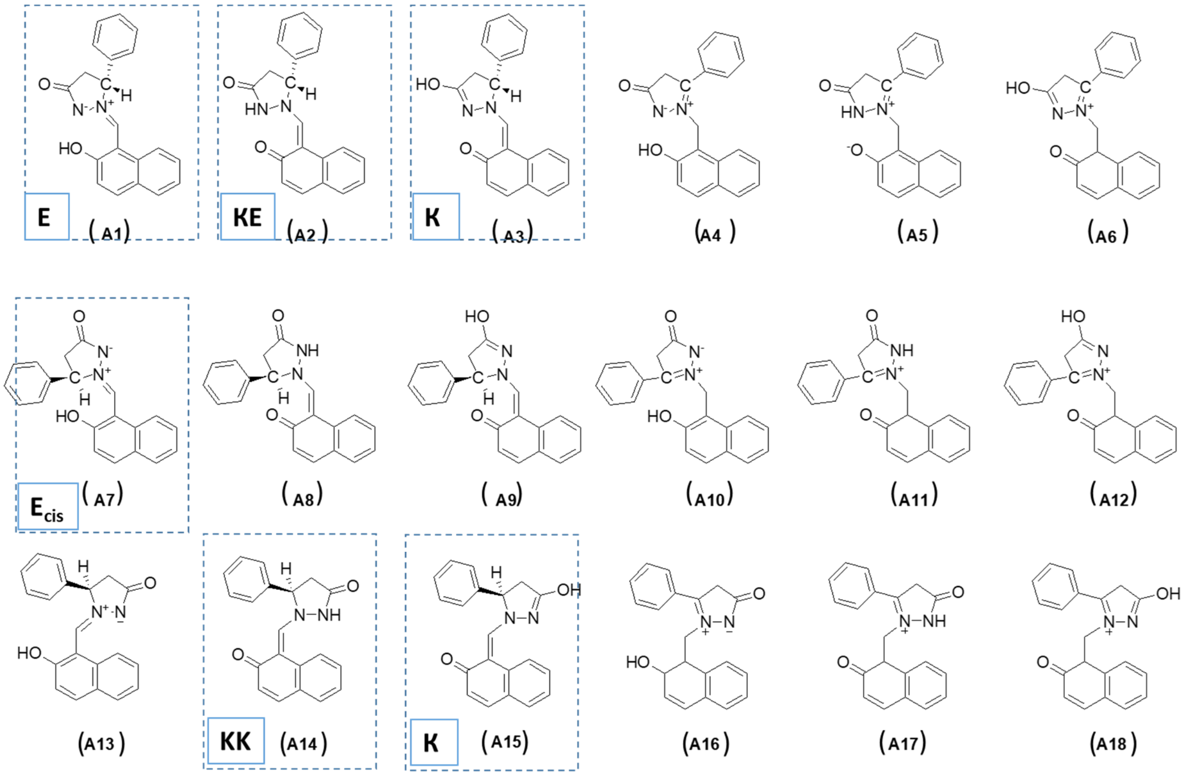

Figure S1. Chemical structures considered as initial geometries for the studied compound **A**. Those used for sketching the PES are circled in blue. Their relation to the PES is denoted with the widely accepted designation **E**, **KE**, **KK**, and **K**.

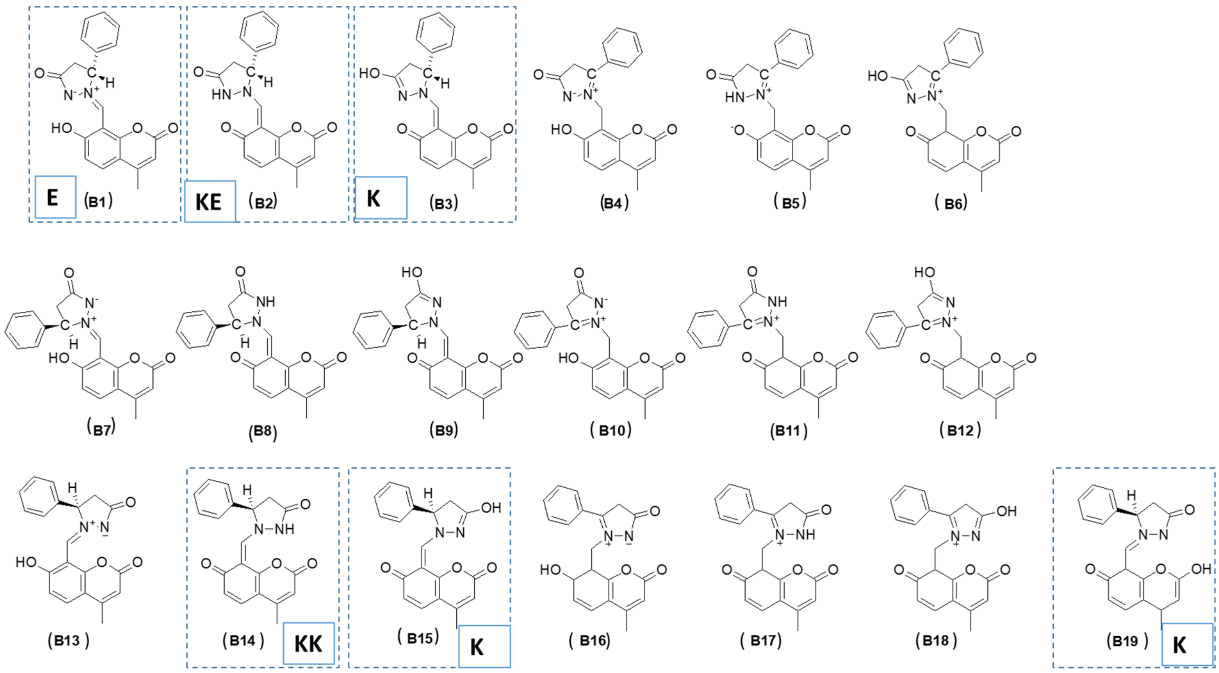

Figure S2. Chemical structures considered as initial geometries for the studied compound **B**. Those used for sketching the PES are circled in blue. Their relation to the PES is denoted with the widely accepted designation **E**, **KE**, **KK**, and **K**.

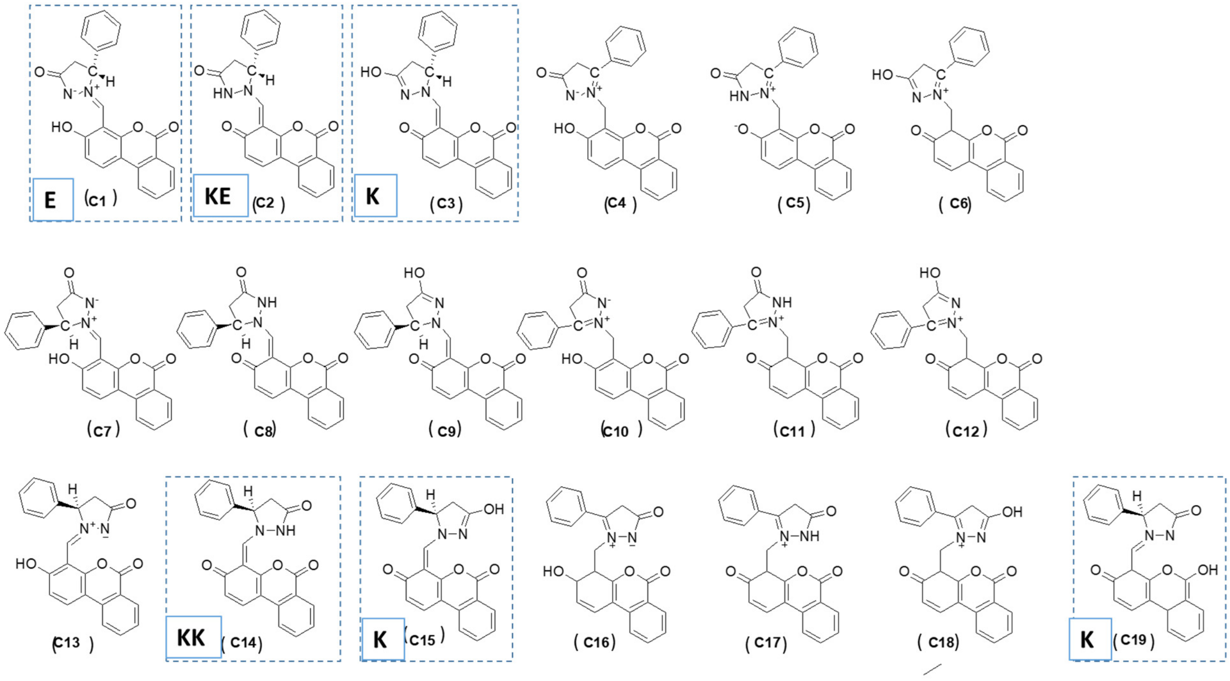

Figure S3. Chemical structures considered as initial geometries for the studied compound **C**. Those used for sketching the PES are circled in blue. Their relation to the PES is denoted with the widely accepted designation **E**, **KE**, **KK**, and **K**.

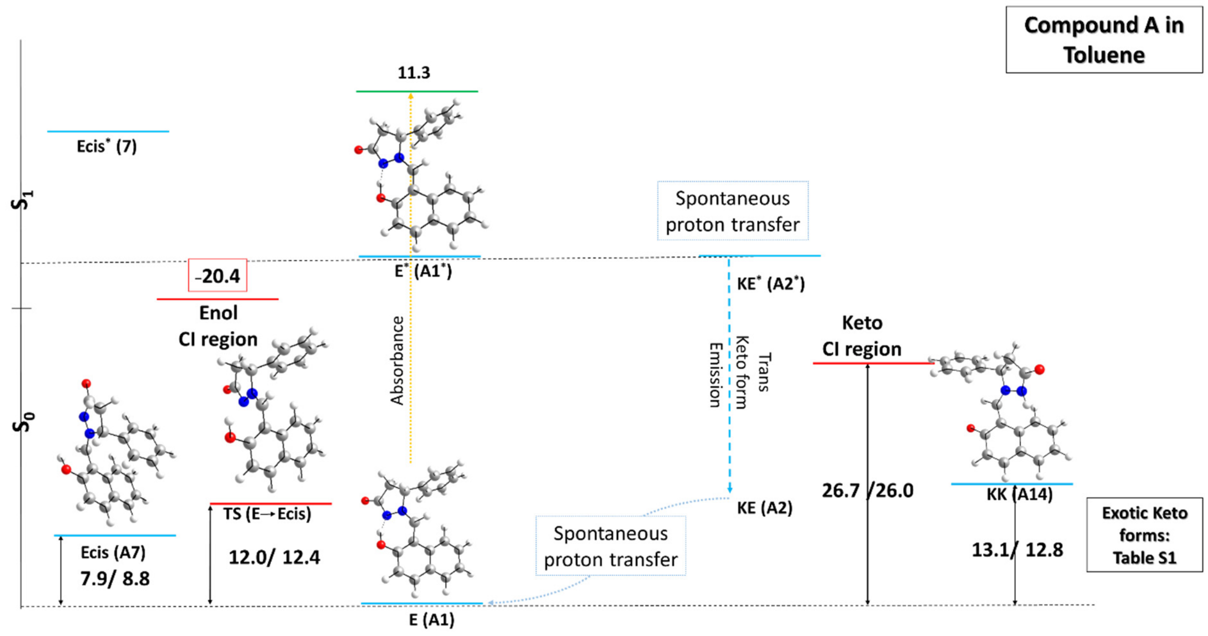

Figure S4. Sketch of the potential energy surface (PES) of model compound **A** (presented as R stereoisomer) in toluene in the ground (M062X/TZVP) and in the first singlet excited (CAM-B3LYP/TZVP) states. The stationary tautomeric forms are indicated in blue, the transition states—in red, the Frank-Condon states—in green. The numerical values present relative energy ΔE/relative Gibbs energy ΔG given in kcal mol^{-1} units. ΔE and ΔG values in the ground and the first singlet excited states are calculated in respect to **E** and **KE***, respectively.

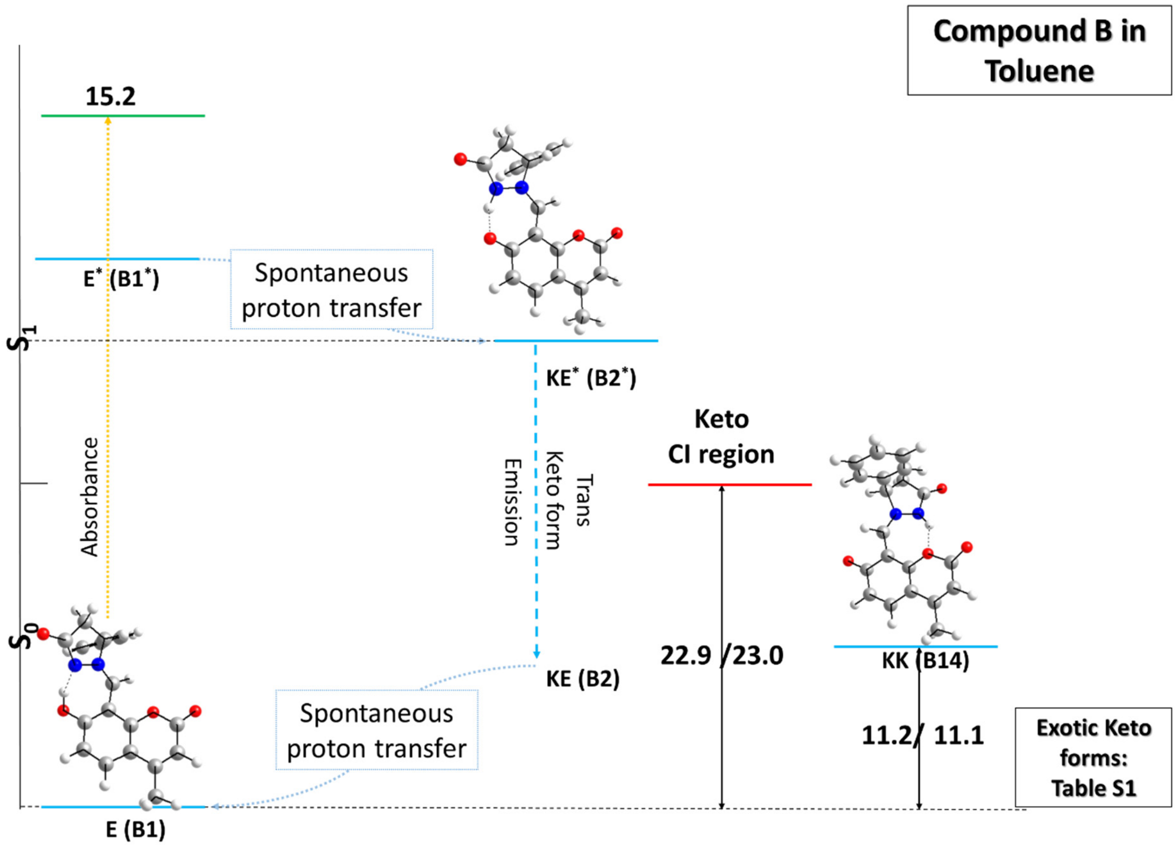

Figure S5. Sketch of the potential energy surface (PES) of model compound **B** (presented as R stereoisomer) in toluene in the ground (M062X/TZVP) and in the first singlet excited (CAM-B3LYP/TZVP) states. The stationary tautomeric forms are indicated in blue, the transition states—in red, the Frank-Condon states—in green. The numerical values present relative energy ΔE/relative Gibbs energy ΔG given in kcal mol^{-1} units. ΔE and ΔG values in the ground and the first singlet excited states are calculated in respect to **E** and **KE***, respectively.

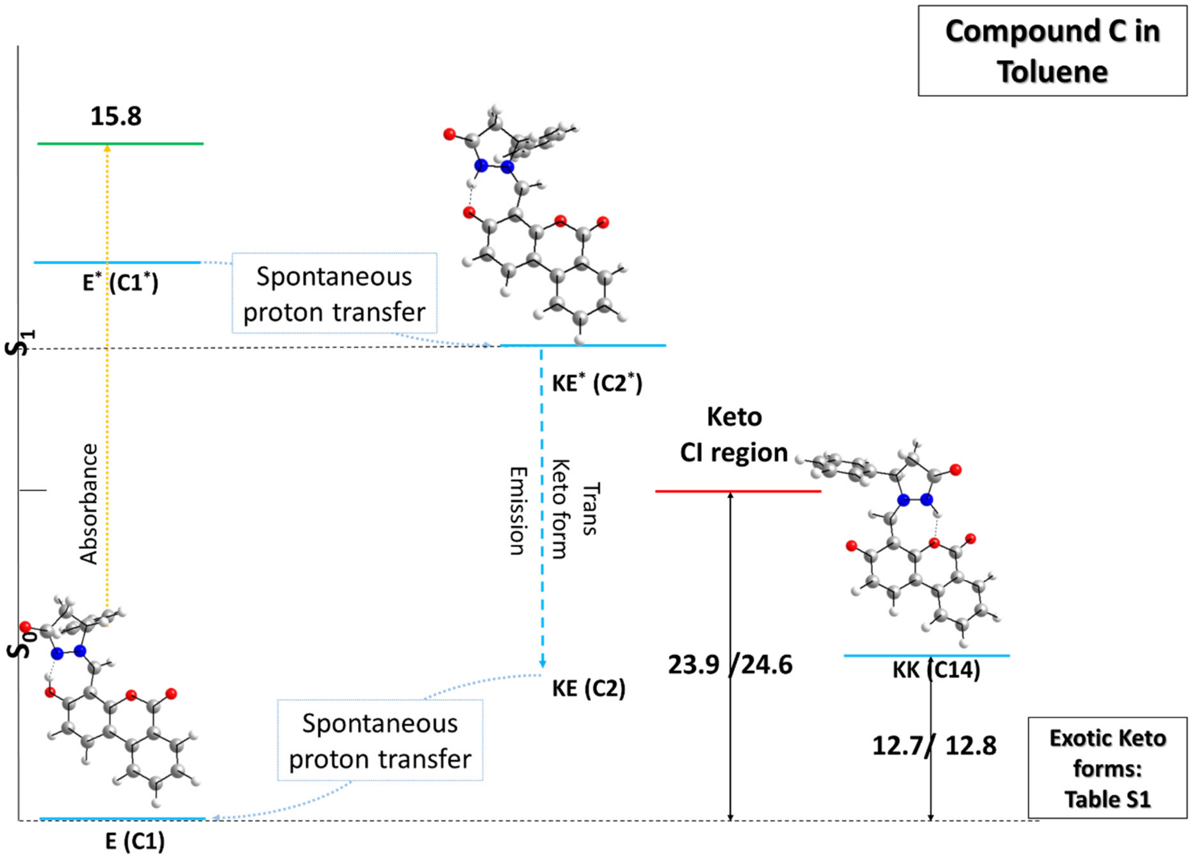

Figure S6. Sketch of the potential energy surface (PES) of model compound **C** (presented as R stereoisomer) in toluene in the ground (M062X/TZVP) and in the first singlet excited (CAM-B3LYP/TZVP) states. The stationary tautomeric forms are indicated in blue, the transition states—in red, the Frank-Condon states—in green. The numerical values present relative energy ΔE/relative Gibbs energy ΔG given in kcal mol^{-1} units. ΔE and ΔG values in the ground and the first singlet excited states are calculated in respect to **E** and **KE***, respectively.

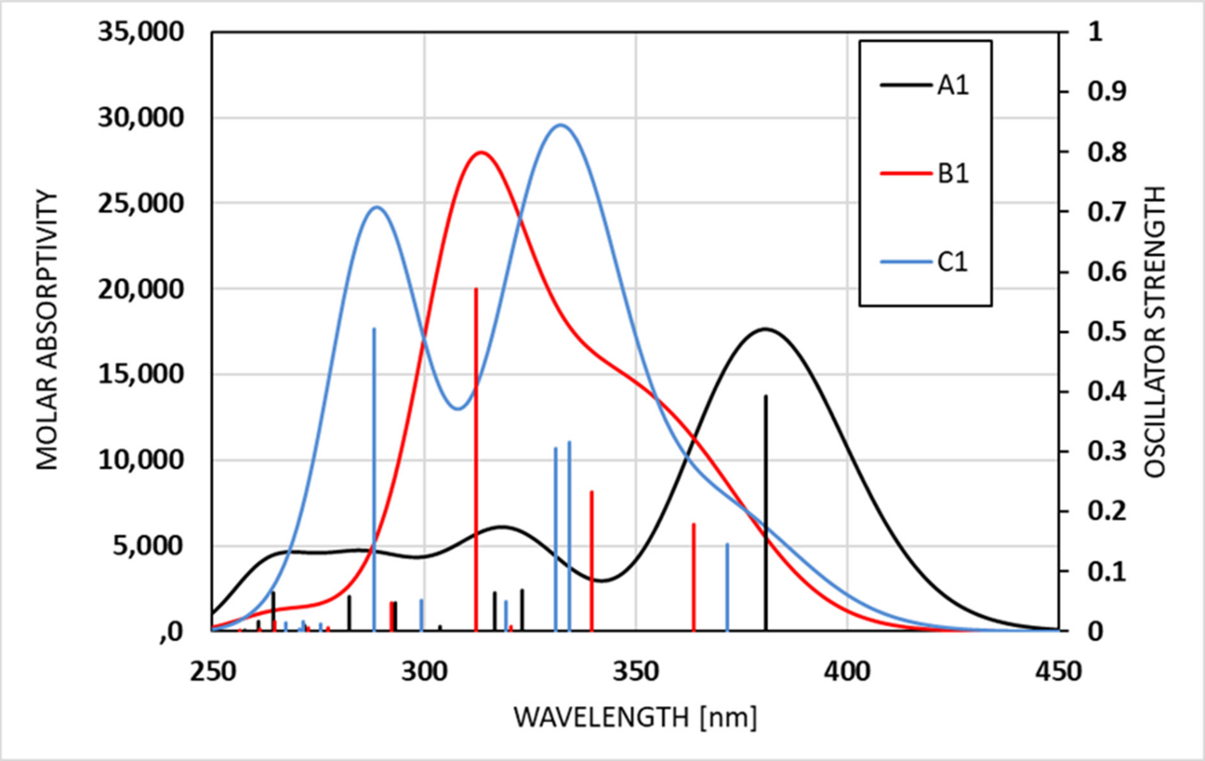

Figure S7. TDDFT simulated absorption spectra of the compounds under study, enol structures **A1** (black), **B1** (red) and **C1** (blue) in toluene. The vertical lines indicate the oscillator strengths of the corresponding transitions.

Table S1. Calculated ΔE/ΔG values in kcal mol^{-1} units at the M062X/TZVP theoretical level for obtaining some intriguing/ exotic keto forms in toluene.

Forms (see Figures S1 to S3)		Optimized geometry	α/β [º]	ΔE	ΔG
Compound **A**	**A3**	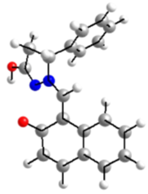	-33.0/ -22.8	18.6	18.1
	**A15**	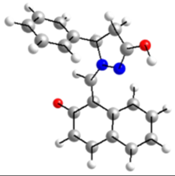	162.7/ -17.2	16.7	16.3
	**A19**	-	-	-	-
Compound **B**	**B3**	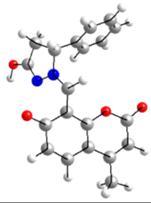	-31.4/ -16.5	18.7	19.1
	**B15**	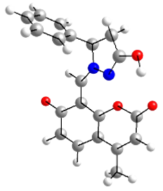	157.7/ -12.4	17.4	17.1
	**B19**	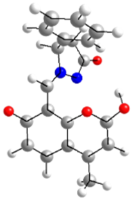	-153.9/ 1.2	27.9	27.1
Compound **C**	**C3**	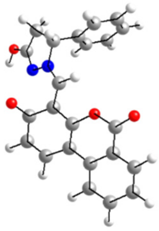	-31.1/ -17.2	19.4	19.6
	**C15**	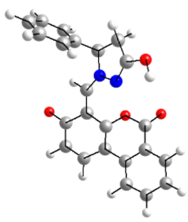	158.9/ -11.5	18.3	18.1

	**C19**	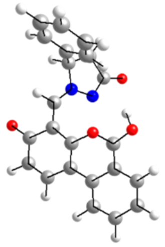	-157.5/ -0.3	36.8	36.8

Table S2. Calculated and experimental (X-ray) parameters for the preferred enol tautomers of compounds **A**-**C** (gas-phase optimized).

		**A1**				
	Parameter	Calculated[S1]	Experimental[S2]	this work	**B1**	**C1**
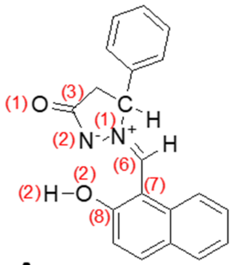	Bond	*d*/Å				
**A**	C(3)-O(1)	1.214	1.226	1.206	1.205	1.205
	C(3)-N(2)	1.373	1.366	1.370	1.372	1.373
	N(1)-N(2)	1.357	1.378	1.351	1.349	1.347
	N(1)-C(6)	1.307	1.297	1.293	1.291	1.291
	C(6)-C(7)	1.444	1.452	1.444	1.449	1.449
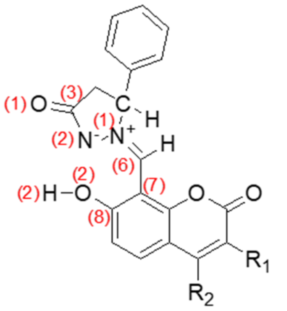	C(7)-C(8)	-	-	1.396	1.414	1.413
	C(8)-O(2)	1.331	1.349	1.327	1.320	1.323
	O(2)-H(2)	1.015	0.929	1.016	1.023	1.019
	H(2)…N(2)	1.590	1.651	1.589	1.562	1.574
	Dihedral angle	ω/deg				
	Fragment I/	34.0	39.6	37.3	31.3	28.6
	Fragment II					
**B:** R_1 = H, R_2 = CH_3<br>**C:** R_1 + R_2 = C_4H_4	Fragment I/Ph	88.1	81.4	71.6	68.8	79.2

Table S3. Calculated excitation energies (E), wavelengths (λ), oscillator strengths (f), and dominant orbital contributions for the low-lying excited states of the preferred enol forms of compounds **A**–**C** in toluene, obtained using TDDFT at the B3LYP/TZVP level of theory. Only excited states with oscillator strengths ≥ 0.1 are reported.

Compound		Transition	Eigenvectors	E, eV	λ, nm	f
**A1**	Excited state 1	83 -> 84 (HOMO -> LUMO)	0.69958	3.2567	381	0.3914
**B1**	Excited state 1	90 -> 92	-0.20225	3.4080	364	0.1773
		91 -> 92 (HOMO -> LUMO)	0.64288			
		91 -> 93	-0.18793			
	Excited state 2	90 -> 92 (HOMO-1 -> LUMO)	0.59753	3.6504	340	0.2331
		91 -> 92	0.25641			
		91 -> 93	0.25105			
	Excited state 4	89 -> 92	-0.11014	3.9709	312	0.5709
		90 -> 92	-0.29254			
		91 -> 93 (HOMO -> LUMO+1)	0.61688			
**C1**	Excited state 1	99 -> 101	-0.15399	3.3354	372	0.1448
		100 -> 101 (HOMO -> LUMO)	0.66810			
		100 -> 102	0.12671			
	Excited state 2	98 -> 101	-0.10931	3.7077	334	0.3153
		99 -> 101 (HOMO-1 -> LUMO)	0.61763			
		100 -> 101	0.18891			
		100 -> 102	-0.23267			
	Excited state 3	99 -> 101	0.23441	3.7442	331	0.3050
		99 -> 102	-0.10404			
		100 -> 102 (HOMO -> LUMO)	0.64199			
	Excited state 6	99 -> 102	-0.15623	4.2998	288	0.5040
		100 -> 103 (HOMO -> LUMO+1)	0.66937			

References

S1. Popova, O. S. *et al.* Benzenoid-quinoid tautomerism of azomethines and their structural analogs 56. Azomethine imines, derivatives of salicylic and 2-hydroxynaphthoic aldehydes. *Russ. Chem. Bull.* **65**, 648–653 (2016).

S2. Nikolaeva, O. G., Popova, O. S., Dubonosova, I. V, Karlutova, O. Y. & Dubonosov, A. D. Spectral-Luminescent and Ionochromic Properties of Azomethine Imine-Coumarin Conjugates. **92**, 841–849 (2022).
